# Recent Advances in Neurogenic Small Molecules as Innovative Treatments for Neurodegenerative Diseases

**DOI:** 10.3390/molecules21091165

**Published:** 2016-09-01

**Authors:** Clara Herrera-Arozamena, Olaia Martí-Marí, Martín Estrada, Mario de la Fuente Revenga, María Isabel Rodríguez-Franco

**Affiliations:** 1 Instituto de Química Médica, Consejo Superior de Investigaciones Científicas (IQM-CSIC), C/Juan de la Cierva 3, Madrid 28006, Spain; clherrer@iqm.csic.es (C.H.-A.); olaia.marti.mari@gmail.com (O.M.-M.); mhestradav@iqm.csic.es (M.E.); 2 Department of Physiology and Biophysics, School of Medicine, Virginia Commonwealth University, Richmond, VA 23298, USA; mario.delafuenterevenga@vcuhealth.org

**Keywords:** adult neurogenesis, serotonin system, melatonin receptors, Wnt/β-catenin pathway, sigma receptors, nicotinamide phosphoribosyltransferase, nuclear erythroid 2-related factor

## Abstract

The central nervous system of adult mammals has long been considered as a complex static structure unable to undergo any regenerative process to refurbish its dead nodes. This dogma was challenged by Altman in the 1960s and neuron self-renewal has been demonstrated ever since in many species, including humans. Aging, neurodegenerative, and some mental diseases are associated with an exponential decrease in brain neurogenesis. Therefore, the controlled pharmacological stimulation of the endogenous neural stem cells (NSCs) niches might counteract the neuronal loss in Alzheimer’s disease (AD) and other pathologies, opening an exciting new therapeutic avenue. In the last years, druggable molecular targets and signalling pathways involved in neurogenic processes have been identified, and as a consequence, different drug types have been developed and tested in neuronal plasticity. This review focuses on recent advances in neurogenic agents acting at serotonin and/or melatonin systems, Wnt/β-catenin pathway, sigma receptors, nicotinamide phosphoribosyltransferase (NAMPT) and nuclear erythroid 2-related factor (Nrf2).

## 1. Introduction

Regenerative medicine is one of the most innovative therapeutic approaches for the treatment of different pathologies. Undoubtedly, it will be a mainstay of the future medicine, although nowadays there are still several obstacles to overcome. One of these challenges is the development of therapies for the central nervous system (CNS). Efficient regeneration of damaged tissues by an accident (e.g., trauma, stroke), by neurodegenerative diseases (e.g., AD, Parkinson) or psychiatric conditions (e.g., mood disorders, anxiety, major depression) would be a great advance to achieve the definitive cure for these diseases [1]. Indeed, the discovery of the existence of neural stem-cells (NSCs) niches in the adult human brain opens the door to the development of small molecules as restorative therapies for such pathologies. Ideally, such medicines should be taken orally and must reach the brain for promoting endogenous auto-repair processes. This strategy is simpler than the implant of pluripotent cells and advantageously avoids the ethical dilemma of the employment of human embryonic NSCs [2].

The generation of new neuronal cells in the brain of vertebrates is an essential process throughout the lifetime of the individual. Although for a long time, neurogenesis was thought to be restricted to the first stages of embryonic development, this concept was challenged in 1962 when Altman showed the formation of new neurons in the brain of adult rats [3]. In subsequent years, this process was described in adult birds [4], rabbits [5], marsupials [6] and primates [7].

However, the therapeutic potential of neurogenesis only began to gain strength in the 1990s with the discovery of two niches containing self-renewing NSCs in the adult murine brain [8,9,10,11]: the subventricular zone (SVZ), lining the lateral ventricles, and the subgranular zone (SGZ) in the dentate gyrus (DG) of hippocampus [12]. The definitive demonstration of the existence of neurogenic processes in the adult human brain was made by Eriksson et al. in 1998, through the examination of post-mortem hippocampal tissues from patients who had been treated with the thymidine analogue bromodeoxyuridine (BrdU) [13], which is commonly used in the detection of proliferating cells in living tissues [14]. These studies demonstrated that the hippocampus is able to develop and integrate new neuronal bodies during adult life, beyond the embryonic development [15].

Embryonic and adult neurogenesis share many transcriptional regulators but display significant differences in their modulation. During the early development, neurogenesis is vast and occurs in a coordinated manner to generate the CNS as the leading machinery of the whole organism, whereas in adulthood neurogenesis is restricted to certain neurogenic niches. Adult NSCs have additional and more advanced modulatory mechanisms, by means of which NSCs can supply specific types of neurons to limited areas of the CNS [16,17,18]. The quantitative differences between embryonic and adult neurogenesis reveal that the post-natal homeostatic function of the brain is rather conservative and does not require major renovations of the neural network for its normal functioning [17]. However, under ischemic conditions, it has been shown that neurogenesis occurs beyond DG and SVZ in adult rats by activation of neuronal progenitor cells in the neocortical layer 1 [19]. Thus, it could tentatively differentiate between a physiological adult neurogenesis in the DG and SVZ and a restorative neurogenesis in larger regions of the CNS, triggered under pathological conditions derived from hypoxic episodes.

Adult neurogenesis is sequential: activation of quiescent NSCs and proliferation, migration to different areas of the CNS, differentiation and maturation to specific cell types, and integration in the brain circuitry [20]. In lower vertebrates the neurogenic process is widely extended in the CNS, whereas in animals with higher neuronal complexity is restricted to the few above-mentioned regions, where the multipotent NSCs generate new neurons and glia cells throughout the individual’s life [21].

The hippocampus is a part of the limbic system that plays important roles in the consolidation of information from short-term to long-term memory [22]. Although the relevance of neurogenesis in the adult hippocampus remains to be determined, some reports link it to learning processes and memory integration [23,24]. Neurogenesis in the DG is dynamic and sensitive to physiological, pathological and pharmacological stimuli [25]. For instance, aging, neurodegeneration, and some mental diseases are associated with an exponential decrease in hippocampal neurogenesis [24]. Therefore, the controlled pharmacological stimulation of the endogenous NSCs from neurogenic niches might counteract the age-related loss of memory and cognitive deterioration in pathological conditions [26].

Since the discovery of the adult neurogenesis, much research effort has been devoted to study its mechanisms and implications in healthy and pathological conditions. Some elucidated neurogenic mechanisms involve neurotransmitters (such as dopamine, glutamate, and serotonin), hormones (such as thyroid hormones and melatonin), signalling pathways (Notch, Wnt/β-catenin, NAMPT-NAD, etc.), transcription factors (Sox-2, the orphan nuclear receptor TLX, Nrf2, etc.), growth factors (brain-derived neurotrophic factor BDNF, insulin-like grow factor-1, fibroblast growth factor 2, etc.) and epigenetic factors (reviewed in [27]). The above findings have led to the discovery of a significant amount of small-molecules able to take part in neuronal renewal and plasticity [28,29,30,31,32]. As an example, in the last five years around 700 reviews on “adult neurogenesis” have been published, as countered from PubMed. The present review only modestly attempts to have a glance in some recent advances in neurogenic small molecules acting at serotonin and/or melatonin systems, Wnt/β-catenin pathway, sigma receptors, nicotinamide phosphoribosyltransferase (NAMPT) and nuclear erythroid 2-related factor (Nrf2).

## 2. Role of Serotonergic System in Neurogenesis

**Figure 1:**
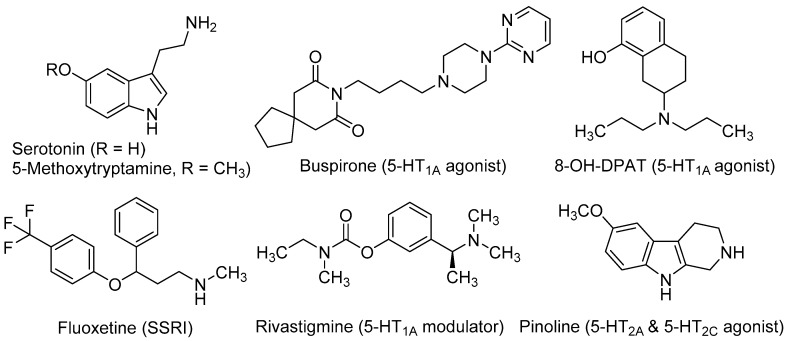
Serotonergic ligands with neurogenic properties.

Serotonin (5-hydroxytryptamine, 5-HT, Figure 1) is crucial for the normal brain development and plays an important role in the CNS over lifetime [33]. This neurotransmitter is involved in almost every adaptive response, such as appetite and mood, and in a great number of cognitive functions. Up to 15 subtypes of serotonin receptors (5-HTRs) mediate the serotonergic tone, both in the central and peripheral nervous system [34]. In relation to neurogenic processes, several studies demonstrated the involvement of serotonergic system in NSC’s proliferation and maturation to a neural phenotype. Brezun and Daszuta showed that serotonin deficits in adult rats are associated with decreased adult neurogenesis in the DG and SVZ [35]. It has been demonstrated its necessity in synaptogenesis [36] and more recently, its importance in developing-axon guidance [37].

In in vitro experiments, Benninghoff et al. found that serotonin depletion hinders the survival and proliferation of NSC-neurospheres derived from adult mouse hippocampus. By selectively antagonizing different 5-HTRs, these authors demonstrated that the neurogenic actions of serotonin in NSCs were mediated by the 5-HT_1A_ subtype and, to a lesser extent, through the 5-HT_2C_R [38]. These findings, together with the observation that increased levels of serotonin have positive effects on neurogenesis and axonal growth, has encouraged the study of agonists at several 5-HTR subtypes as well as selective serotonin reuptake inhibitors (SSRIs) as neurogenic agents, both in vitro and in vivo models with interesting results.

Several in vivo studies demonstrated the implication of the 5-HT_1A_ receptor subtype in neurogenic processes. Grabiec et al. studied the effect of the 5-HT_1A_ agonists buspirone (8-[4-(4-pyrimidin-2-ylpiperazin-1-yl)butyl]-8-azaspiro[4,5]decane-7,9-dione) and 8-OH-DPAT (8-hydroxy-2-(di-*n*-propylamino)tetralin, Figure 1), in comparison with the 5-HT_1A_ antagonist WAY100635 (*N*-[2-[4-(2-methoxyphenyl)-1-piperazinyl]ethyl]-*N*-(2-pyridyl)cyclohexanecarboxamide) in *Monodelphis domestica* (a small South American marsupial, also named opossum). Compounds were injected during seven successive days and animals were sacrificed two months later. Authors found that the 5-HT_1A_ agonists (buspirone and 8-OH-DPAT) increased the number of newly generated neuronal cells, whereas the antagonist WAY100635 reduced them. Moreover, these 5-HT_1A_ agonists stimulated the differentiation of NSCs mainly into neurons (55%–76%), while a lower proportion was transformed into astroglia (6%–12%) [39]. Behavioural experiments in the opossums treated with 5-HT_1A_ agonists demonstrated that the major number of new neurons correlated with better cognitive performance in a test for detecting hidden food based on olfactory perception [40].

Fluoxetine (*N*-methyl-3-phenyl-3-[4-(trifluoromethyl)phenoxy]propan-1-amine, Figure 1), better known by its commercial name Prozac^®^, is a SSRI used as antidepressant for the treatment of major depression, obsessive-compulsive disorder, and bulimia, among other psychiatric diseases. Recent studies conducted in a mouse model of Down syndrome showed that treatment with fluoxetine during the embryonic period rescued overall brain development and that neonatal treatment induced full recovery of hippocampal neurogenesis, dendritic development, connectivity and hippocampus-dependent memory [41,42]. The effects of either the pre- or early postnatal treatments were retained in adolescence and adult life stages, showing that the early treatment with fluoxetine enduringly restores cognitive impairment and prevents early signs of AD-like pathology [42].

Rivastigmine [(*S*)-3-[1-(dimethylamino)ethyl]phenyl *N*-ethyl-*N*-methylcarbamate, Figure 1] is used to treat mild to moderate dementia in AD. It acts as a non-competitive inhibitor of both acetylcholinesterase (AChE) and butylcholinesterase (BuChE), which improves cognitive functions by increasing acetylcholine levels at the synaptic cleft. Moreover, rivastigmine ameliorates depression-like symptoms in patients by mechanisms that are not fully understood [43]. Recently, it has been observed that after administration of rivastigmine to olfactory bulbectomized (OBX) mice, hippocampal neurogenesis increased along with enhanced levels of protein kinase B (PKB), and extracellular signal-regulated kinase (ERK) phosphorylation. The ability of rivastigmine to rescue 5HT_1A_R levels in hippocampus appears to underlie such effects [44,45].

Pinoline (6-methoxy-1,2,3,4-tetrahydro-9*H*-pyrido[3,4-*b*]indole, Figure 1) is a tricyclic β-carboline and a conformationally-restricted analogue of the serotonin derivative 5-methoxy-tryptamine (5-MeOT). Pinoline is a modest inhibitor of monoaminoxidase A and a potent oxygen radical scavenger. It also activates 5-HTRs, being a full agonist at the 5-HT_2C_ subtype and a partial agonist at the 5-HT_2A_R. Trace concentrations of pinoline (10 nM) were found to stimulate early differentiation and neuronal maturation of primary cultures of NSC derived from the rat SVZ [46]. Its potent neurogenic properties in vitro correlate with its serotonergic agonism.

## 3. Melatonergic System Role in Neurogenesis

**Figure 2:**
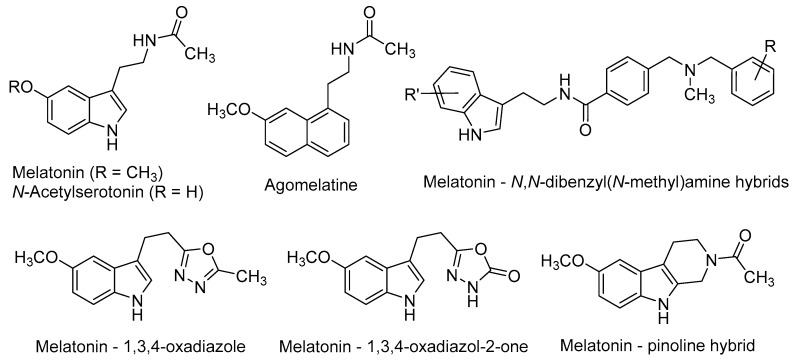
Neurogenic agents related to melatonin.

Melatonin [*N*-(2-(5-methoxy-1*H*-indol-3-yl)ethyl)acetamide, Figure 2] is a neurohormone produced primarily in the pineal gland. Two G-protein-coupled receptors (GPCRs), namely MT_1_ and MT_2_ (MT_1_R and MT_2_R) [47,48] mediate most of the vast physiological and pharmacological actions of melatonin, such as the regulation of circadian and seasonal rhythms’, immune and endogenous antioxidant systems, among others [49]. Melatonin is a potent free-radical scavenger and a wide-spectrum antioxidant [50,51], improves mitochondrial energy metabolism [52], decreases neurofilament hyperphosphorylation and plays a neuroprotective role against beta-amyloid peptide (Aβ) [53]. Moreover, the long-term oral administration of melatonin improves learning and memory in the AD-model APP/Ps1 transgenic mouse [54].

Melatonin plasma levels decline along with age in a similar manner as the neuronal self-renewal rate does [55]. Whether both phenomena are related or not is still unclear, although melatonin has been reported to positively modulate neurogenesis, as recently reviewed by several authors [56,57,58,59].

Exogenous administration of melatonin increases precursor cell survival in hippocampus of mice, promotes neuronal differentiation [60], and stimulates the maturation and complexity of dendrites in the newly formed neurons [61]. Its chronic administration increases the cell proliferation rate and delays the decline of neurogenesis in the hippocampus of adult mice [62]. These data are in agreement with previous observations in different mice strains that concluded that exogenous melatonin administration delays the onset of poor health states prior to death and ameliorates the process of aging, including a much better cognitive performance [63]. The term ‘Methuselah syndrome’ was appropriately coined to summarize the health-preserving properties of melatonin. Over the past years, many scientific papers have focused on the neurogenic properties of melatonin-related compounds in both in vitro and in vivo murine models [64,65,66].

*N*-Acetylserotonin [*N*-(2-(5-hydroxy-1*H*-indol-3-yl)ethyl)acetamide, Figure 2] is the immediate precursor of melatonin. Until recent years, it was thought to lack any relevant biological activity by itself. However, its distribution in some brain areas where serotonin and melatonin are absent, suggests that it may have other physiological roles [67]. Besides its MT_1_/MT_2_ agonism, *N*-acetylserotonin has been shown to act as a potent agonist at the tyrosine receptor kinase (TrkB) receptor, whereas serotonin and melatonin do not [67,68]. TrkB belongs to a family of tyrosine kinases that regulates neuronal survival and neural plasticity in the mammalian nervous system. TrkB binds the brain-derived neurotrophic factor (BDNF) with high affinity, triggering signalling pathways that regulate long-term potentiation (LTP), neurogenesis and neuronal plasticity [69,70]. TrkB blockade abolished the effects of *N*-acetylserotonin on neural cell proliferation, thus suggesting the key role of TrkB on the positive effects of *N*-acetylserotonin in adult mice neurogenesis [71].

Agomelatine (*N*-[2-(7-methoxynaphthalen-1-yl)ethyl]acetamide, Figure 2), marketed as Valdoxan^®^ for the treatment of major depression in adults, shows a mixed profile (MT_1_/MT_2_ agonism and 5-HT_2C_ antagonism). Agomelatine was found to positively affect all stages of neurogenesis in rat hippocampus under normal or stress conditions [72,73]. Banasr et al. found that chronic administration of agomelatine (21 days) increased NSCs proliferation and maturation in the hippocampal DG, in contrast with acute (4 h) or subchronic (7 days) treatments [74]. In an effort to elucidate the neurogenic mechanisms of agomelatine, Soumier et al. found that this drug increased the ratio of mature vs. immature neurons and also promoted neurite outgrowth, probably through the BDNF up-regulation subsequent to MT_1_/MT_2_ stimulation [75]. 

Recently, several melatonin-based compounds have been tested in primary hippocampal NSCs of adult rats. A family of melatonin–*N*,*N*-dibenzyl(*N*-methyl)amine hybrids (Figure 2), showing a balanced multifunctional profile covering antioxidant, cholinergic and neuroprotective properties, was found to stimulate NSCs proliferation and the subsequent maturation into a neuronal phenotype. Some hybrids protected neuronal cells against mitochondrial oxidative stress and were 2-fold more potent than melatonin as neurogenic agents [76].

*N*-Acetyl bioisosteres of melatonin were recently obtained by replacing the acetamido group of the natural product by a series of reversed amides and azoles (Figure 2). Several of these melatonin bioisosteres promoted differentiation of rat neural stem cells to a neuronal phenotype in vitro, in some cases with greater potency than melatonin itself. In the case of the melatonin-based compounds derived from 1,3,4-oxadiazole and 1,3,4-oxadiazol-2-one, their neurogenic effects were almost completely blocked by luzindole [*N*-(2-(2-benzyl-1*H*-indol-3-yl)ethyl)acetamide], a non-selective MT_1_R/MT_2_R antagonist. These results evidenced the key implication of melatonergic receptors in the neurogenic properties of these compounds, although the participation of additional signalling pathways cannot be excluded [77].

Melatonin and pinoline hybrids with β- and γ-carboline scaffolds retained the neurogenic properties of the parent compounds in an in vitro model of neural stem cell differentiation. The orthodox melatonin–pinoline hybrid (Figure 2) displayed partial melatonergic agonism, with a slight preference for the MT_2_R subtype, and promoted early differentiation and neuronal maturation in in vitro experiments. These effects are likely mediated by melatonergic stimulation [46].

## 4. Effects of the Wnt/β-Catenin Pathway in Neurogenesis

The canonical Wnt/β-catenin pathway displays important roles in both embryonic CNS-development and adult brain self-repairing processes [78]. During embryonic growth, Wnt signalling is essential for the appropriate formation of hippocampus and cortex [79], whereas in the adult brain it regulates neurogenesis and synaptic plasticity [80]. 

Modulation of adult neurogenesis by Wnt pathway was demonstrated in vivo by Lie et al. Blockage of the Wnt signalling reduced NSC proliferation and maturation in the SGZ in rats, whereas Wnt activation improved neurogenic processes [81]. Moreover, Jessberger et al. revealed that Wnt-mediated neurogenesis contributes to hippocampal memory functions, as Wnt blockade produced impaired long-term spatial memory and object recognition in adult rats [82]. 

The enzyme glycogen synthase kinase-3β (GSK-3β) is an important component of the Wnt/β-catenin pathway, which phosphorylates β-catenin and modifies the signalling cascade that finally modulates adult neurogenesis. Interestingly, Eom and Jope reported a drastic neurogenic drop in the GSK-3α/β knock-in mouse, compared with the wild-type [83]. Thus, GSK-3β overexpression was found to provoke neuronal death and to diminish the NSC proliferative rate in the hippocampal DG by a depletion of neurogenic niches [84]. Therefore, the inhibition of GSK-3β and subsequent activation of Wnt/β-catenin signalling pathway could promote hippocampal neurogenesis, as shown in the following examples.

**Figure 3:**
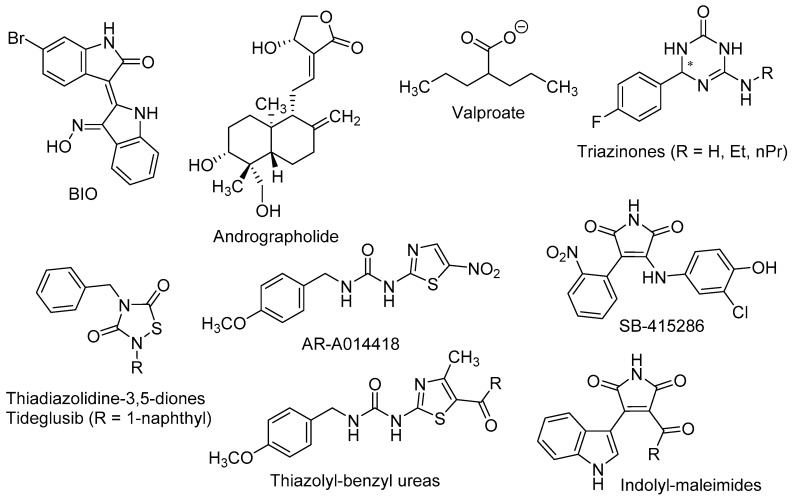
Neurogenic molecules acting at the Wnt/β-catenin pathway.

Wexler et al. found that the mood stabilizer lithium, also known as a potent GSK-3β inhibitor, induced the proliferation and differentiation of adult hippocampal progenitor cells in primary cell cultures. The positive effects of lithium on neurogenic processes were found to be independent of inositol depletion, but linked to GSK-3β inhibition and β-catenin activation [85]. Dastjerdi et al. demonstrated that 6-bromoindirubin-3′-oxime (BIO, Figure 3), a specific GSK-3β inhibitor and Wnt activator, increased the expression of the early neurogenic marker β-III-tubulin in unrestricted somatic stem cells, pointing out that GSK-3β inhibition enhanced their neural differentiation [86].

The in vivo confirmations of the above results were made in murine models of stress, depression, and toxic conditions, as well as in models of neurodegenerative diseases, such as AD. In pre-pubertal and adult rats subjected to chronic-mild-stress, Silva et al. observed that lithium treatment improved behavioural, hormonal, and neurogenic turnover in the SVZ [87]. These authors also reported that inhibition of GSK-3β by the thiazolyl-benzyl urea AR-A014418 [1-(4-methoxybenzyl)-3-(5-nitrothiazol-2-yl)urea, Figure 3] yielded higher levels of the presynaptic protein synapsin-I (a known target of GSK-3β), which in turn alleviated stress effects and neurogenic depletion. 

Valproate (2-propylpentanoate, Figure 3) is a well-known drug, used to the treatment of epilepsy, bipolar disorders and migraine. Despite its complex polypharmacology, some studies reported that valproate promotes β-catenin pathway trough GSK-3β inhibition [88] and more recently, that it recovered the proliferation rate of neural precursor cells in the DG in a rat model of depression [89].

Fujimura and Usuki showed that low concentration of methylmercury (MeHg, 10 nM) suppressed the proliferation of neural progenitor cells of rat cortical tissues. These effects were reversed by GSK-3β inhibitors, such as lithium and the maleimide derivative SB-415286 [3-((3-chloro-4-hydroxyphenyl)amino)-4-(2-nitrophenyl)-1*H*-pyrrole-2,5-dione, Figure 3], by a mechanism that involved the blockade of cyclin E degradation [90]. 

Fiorentini et al. reported that lithium treatment improved hippocampal neurogenesis and cognition functions in the double transgenic CRND8 mice, an AD-model that develop early plaque formation [91]. Andrographolide (3-[2-(decahydro-6-hydroxy-5-hydroxymethyl-5,8*a*-dimethyl-2-methylene-1-napthalenyl)ethylidene]dihydro-4-hydroxy-2(3*H*)-furanone, Figure 3), a diterpenoid isolated from *Andrographis paniculata*, has been intensely studied in cell signalling pathways and shows neuroprotective effects in different models of oxidative damage [92]. In the APPswe/PS1ΔE9 mouse model of AD, this terpenoid improved the consolidation of LTP against the damage induced by Aβ oligomers, most likely by inhibiting GSK-3β [93]. Moreover, andrographolide promoted proliferation of neural progenitor cells and the generation of new neurons in the adult hippocampus of wild-type and APPswe/PS1ΔE9 mice, by stimulation of the Wnt/β-catenin signalling pathway [94].

Using rat NSCs neurosphere cultures, several GSK-3β inhibitors were tested as neurogenic agents, namely thiadiazolidine-3,5-diones, thiazolyl-benzyl ureas and indolyl-maleimides [95]. These diverse small molecules are related to tideglusib [4-benzyl-2-(naphthalen-1-yl)-1,2,4-thiadiazolidine-3,5-dione], which reached phase II in clinical trials for AD [96], AR-A014418 and SB-415286, respectively (Figure 3). Morales-García et al. showed that these GSK-3β inhibitors increased the number, migration, and differentiation ability of NSCs in in vitro experiments. Moreover, in adult male Wistar rats the oral administration of tideglusib positively affected the neuronal proliferation in SGZ and hilus, dendritic arborisation and neuroblasts’ migration [95]. 

More recently, Prati et al. described triazin-ones and -thiones (Figure 3) as dual inhibitors of both BACE-1 and GSK-3β that also displayed effective neurogenic activities in neurosphere cultures of primary NSCs from rats [97,98]. The most active neurogenic agents were found to be triazinones with R = H, ethyl, and *n*-propyl (Figure 3), which significantly increased the number of cells reaching a mature neuronal state as shown by immunostaining with MAP2.

## 5. Sigma Receptors Role in Neurogenesis

Sigma receptors (σRs) appear to be involved in numerous biological functions, including cell survival. They were originally misclassified as a subtype of opioid receptor in the 1970s, but σRs are currently considered as a unique type of intracellular proteins, different from GPCRs and ionotropic receptors. In humans, σRs and in particular the subtype-1, are expressed in regions of the CNS related to motor, emotional, and cognitive functions: hippocampus, cerebral cortex, substantia nigra, DG of hippocampus and cerebellum [99,100]. Interestingly, adult σ_1_R knockout mice show a depressive-like phenotype and a reduced neurogenic turnover in the DG [101]. Moreover, a significant reduction in the overall levels of the σ_1_R protein has been recently reported in the AD-brains [102] and in the lumbar spinal cord of patients suffering amyotrophic lateral sclerosis (ALS) [103].

It has been also demonstrated that the activation of σ_1_R elicits neuroprotection by different mechanisms: modulation of voltage-dependent calcium channels involved in calcium homeostasis [104]; attenuation of the production of reactive oxygen and nitrogen species, mainly through the modulation of endogenous antioxidant proteins [105,106]; and preservation of mitochondrial function in ischemic stress conditions, by increasing mitochondrial respiration and ATP synthesis [107].

**Figure 4:**
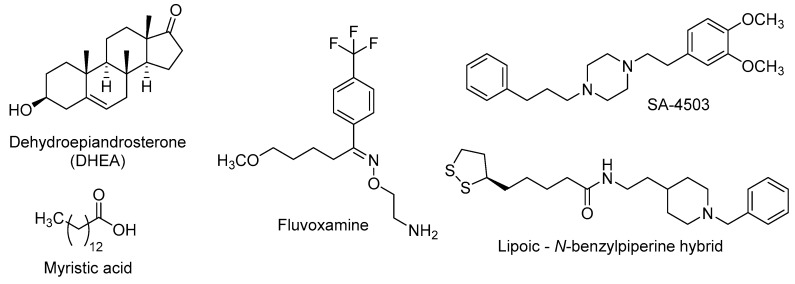
Some σ_1_R agonists with neurogenic properties.

In addition to the above-mentioned neuroprotective properties, activation of σ_1_R has also positive effects on neurogenic processes and dendritic outgrowth. Thus, several σ_1_R agonists of natural origin have been probed, such as DHEA (dehydroepiandrosterone) and myristic acid (tetradecanoic acid), or from pharmaceutical design, such as fluvoxamine (2-{[(*E*)-{5-methoxy-1-[4-(trifluoromethyl)phenyl]pentylidene}amino]oxy}ethanamine), SA-4503 (1-[2-(3,4-dimethoxyphenyl)ethyl]-4-(3-phenylpropyl)piperazine), or lipoic acid-donepezil derivatives (Figure 4).

DHEA is an endogenous neuroactive steroid derived from cholesterol that is secreted by adrenal glands, gonads and CNS-cells, and whose blood levels decrease along age [108,109]. The DHEA age-dependent decline could be linked to the onset of age-related cognitive deficits, such as AD [110], and this fact has prompted its study in several murine models of neurodegenerative diseases. In OBX mice, the σ_1_R stimulation by DHEA ameliorates depressive-like behaviours and cognitive impairments [111] and these effects are associated with improved hippocampal neurogenesis in the DG through the activation of PKB/GSK-3β/β-catenin pathway [112]. DHEA is also found to stimulate dendritic arborisation and neurite outgrowth by increasing the expression of the glial cell-derived neurotrophic factor (GDNF) [113,114], thus promoting the formation of new connections in neuronal plasticity. 

Myristic acid (Figure 4) is a saturated fatty acid, isolated from *Myristica fragrans* nuts, with σ_1_R agonistic properties. Tsai et al. found that σ_1_R activation by myristic acid regulates the correct tau phosphorylation and promotes axon elongation [115].

Since several studies found that adult neurogenesis is impaired in models of depression, a number of antidepressants have been tested as neurogenic agents [116]. Fluvoxamine is a marketed SSRI that also displays a potent agonism at σ_1_R, in the nanomolar range [117]. This antidepressant was found to potentiate nerve-growth factor (NGF)-induced neurite outgrowth in PC12 cells, an effect that was blocked by the σ_1_R antagonist NE-100 [4-methoxy-3-(2-phenylethoxy)-*N*,*N*-dipropylbenzeneethanamine], pointing out the involvement of σ_1_R in the neuroplasticity actions of fluvoxamine [118].

Further research was performed on the neurogenic effects of σ_1_R agonists, namely fluvoxamine and SA-4503, in calcium/calmodulin-dependent protein kinase IV (CaMKIV)-null mice, which exhibit depressive-like behaviours and impaired neurogenesis. The chronic stimulation of σ_1_R by treatment with the above-mentioned σ_1_R agonists increased hippocampal neurogenesis, improving induction and maintenance of hippocampal LTP. Such effects are closely related with the improvement of depressive-like behaviours in CaMKIV-null mice [119].

Recently, a lipoic acid–*N*-benzylpiperine hybrid (Figure 4) was evaluated in primary hippocampal NSCs of adult rats. This nanomolar σ_1_R agonist induced the expression of early neurogenesis markers and also promoted neuronal maturation [120], probably mediated by σ_1_R stimulation.

## 6. Role of NAMPT-NAD in Neurogenesis

Nicotinamide phosphoribosyl transferase (NAMPT) is the rate limiting-enzyme in the biosynthesis of nicotinamide adenine dinucleotide (NAD), an important coenzyme in cellular metabolism and mitochondrial energy production. NAMPT is mainly expressed in nucleus and cytoplasm, although it can be also found in the extracellular space. Its wide distribution and its decisive role in mitochondrial biogenesis indicate that NAMPT plays important functions in the cellular metabolism [121]. 

Regarding neurogenesis, NAMPT is particularly important in the proliferation and differentiation of NSCs. Some consequences of NAMPT inactivation or complete ablation include impairment of NSCs’ proliferation and self-renewal, as well as diminished NSC-mediated oligodendrogenesis [122]. Under aging conditions, NAMPT and NAD levels are considerably reduced in hippocampus, along with NSCs. These age-related reductions and their associated serious consequences in mitochondrial metabolism suggest that NAMPT activation could be a good therapeutic intervention to increase survival and function of NSCs [123].

**Figure 5:**
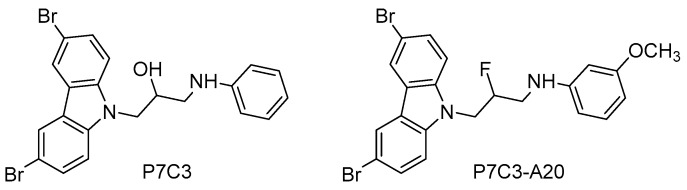
NAMPT activators with neuroprotective and proneurogenic effects.

Recently, a family of NAMPT activators has been described with interesting neuroprotective and proneurogenic effects. The aminopropyl carbazole P7C3 [1-(3,6-dibromo-9*H*-carbazol-9-yl)-3-(phenylamino)propan-2-ol, Figure 5], which was emerged from an in vivo neurogenic screen of 1000 small molecules, improved proliferation and survival of newborn neurons in the hippocampal DG, protected mitochondrial membrane integrity, and ameliorated cognitive decline in aged rats [124]. 

Based on the aminopropyl carbazole scaffold, many P7C3 derivatives were developed (i.e., P7C3-A20, *N*-(3-(3,6-dibromo-9*H*-carbazol-9-yl)-2-fluoropropyl)-3-methoxyaniline, Figure 5) and studied as neurogenic agents in models of acute and chronic neurological diseases, such as traumatic brain injury [125], Down syndrome [126], Parkinson disease [127], and ALS [128]. Mechanistic studies have demonstrated that the neurogenic actions of P7C3 and its derivatives are due to the activation of the NAMPT-NAD cascade [129].

Consequently, there is sufficient evidence supporting the NAMPT-NAD pathway as a valid target in restoring neurogenesis. On the other hand, the potential of this kind of compounds should be further evaluated in order to understand the effects of improving neurogenic processes in acute, chronic and degenerative neurological diseases.

## 7. Role of the Transcription Factor Nrf2 in Neurogenesis

The nuclear erythroid 2-related factor (Nrf2) is a transcription factor that regulates survival genes and the production of antioxidant enzymes. In non-stressed conditions, Nrf2 is mainly located in the cytosol, where its constitutive low levels are strictly controlled by the proteasome [130]. Under pathological conditions (oxidative stress, toxic insults, etc.) the Nrf2 proteolytic degradation is diminished, leading to Nrf2 accumulation in the cytosol and its further translocation to the nucleus. Nuclear Nrf2 binds to the antioxidant response elements (AREs), which are common promoters in many cytoprotective genes, inducing the expression of defence proteins such as NAD(P)H-quinone oxidoreductase 1 (NQO1), glutathione-*S*-transferase, and heme oxygenase-1 (HO-1), among others [131].

Studies in Nrf2-knockout mice have demonstrated that loss of the Nrf2-AREs signalling pathway increases vulnerability to toxic conditions, due to failures in the self-protective responses [132]. Moreover, human epidemiological studies have revealed a link between Nrf2 mutations and the risk of suffering many pathologies, including degenerative diseases [133].

In addition to its pivotal role in the endogenous defence, Nrf2 is an important player in the regulation of neurogenesis. Overexpression of Nrf2 and its downstream genes increased neuronal cell proliferation and differentiation in the human neuroblastoma cell line SH-SY5Y and in rat NSC-derived neurospheres [134,135]. The above data support the use of activators of the Nrf2-AREs signalling pathway, such as curcumin and resveratrol, as neurogenic agents for the treatment of neurological diseases. It is important to note that in addition to the Nrf2 activation, these phytochemicals may trigger other interconnected pathways and, as a result, their mechanisms of action are certainly complex.

**Figure 6:**
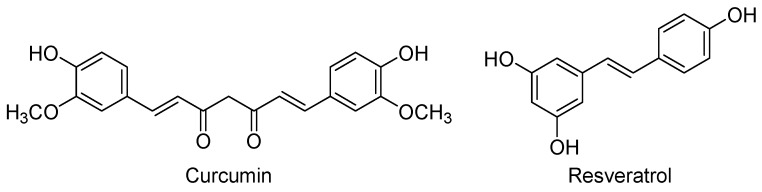
Some activators of the Nrf2-AREs signalling pathway with neurogenic properties.

Curcumin [(1*E*,6*E*)-1,7-bis(4-hydroxy-3-methoxyphenyl)-1,6-heptadiene-3,5-dione] is the major phenolic component of yellow curry, which is extracted from the rhizomes of *Curcuma longa* and is widely used in Asian cooking and traditional medicine (Figure 6). Curcumin is a lipophilic compound that easily penetrates into the CNS and reaches its maximal concentrations in hippocampus, although it also suffers a rapid metabolism and excretion [136]. This low bioavailability has led to the synthesis of curcumin derivatives with improved pharmacokinetics [137,138], or to the development of nano-formulations that considerably prolonged the curcumin’s half-life in cerebral cortex and hippocampus [136].

In in vitro experiments, Kang et al. found that curcumin induced the generation of new neurons, the formation of new synaptic networks and the migration of neural progenitors in brain-derived adult NSCs. Curcumin promoted NSC-differentiation to neurons, but not to astrocytes [139]. In a combination of in vitro and in vivo experiments, Kim et al. have demonstrated that curcumin increases the proliferation of embryonic cortical NSCs in cultures and the number of newly generated cells in the DG of the hippocampus in adult mice. Interestingly, low concentrations of curcumin (500 nM) stimulated the proliferation of embryonic cortical NSCs, whereas concentrations above 10 µM were cytotoxic and inhibited NSC growth [140].

The therapeutic potential of curcumin has been probed in cellular and animal models of different pathologies, including neurodegenerative disorders [137,141]. In a murine AD-model, curcumin inhibited the formation and aggregation of Aβ oligomers and fibrils, disrupted existing amyloid plaques, and partially restored damaged neurites [142].

Based on these interesting findings, curcumin has also tested in humans. In a six-month pilot clinical trial in AD-patients, curcumin (1 g once daily) did not cause side effects; raised plasmatic vitamin E, due to its antioxidant properties; and slightly increased serum Aβ, possibly reflecting the ability of curcumin to disaggregate amyloid deposits in the brain [143].

Resveratrol (*trans*-3,5,4′-trihydroxystilbene, Figure 6) is a natural polyphenol produced by several plants as a defence mechanism against pathogens, such as bacteria and fungi. Red grapes and red wine are rich in this nutraceutical that is believed to be the main promoter of the “French paradox”: a low incidence of coronary heart diseases in people who have a relatively rich diet in cholesterol and saturated fats, but moderate consumption of red wine [144]. Like curcumin, resveratrol can cross the blood-brain barrier to reach its cerebral targets, but it is also rapidly metabolised [145]. To overcome this problem new formulations have been probed, being one of the most recent the nanoencapsulation with chitosan and γ-poly(glutamic acid) that improve the solubility, stability, and bioavailability of resveratrol [146]. Moreover, a current active field is the research of resveratrol-based derivatives with improved pharmacokinetics, some of them in clinical studies for different therapeutic uses [147,148].

The effects of resveratrol on neurogenic processes show a dual pattern depending on the dose, as shown by Kumar et al. using in vitro and in vivo experiments. In cultured embryonic NSC derived from Wistar rats, resveratrol increased cell proliferation at low concentration (10 µM), whereas it exhibited inhibitory effects at high proportions (>20 µM). In adult rats, administration of resveratrol (20 mg/kg body weight) increased the number of new cells in hippocampus [149].

Moreover, Torres-Pérez et al. found that in Balb/C mice, a rodent strain with low baseline levels of adult neurogenesis, the treatment with resveratrol during two weeks increased hippocampal cell survival and NSC proliferation, improving hippocampal plasticity and memory performance [150].

Resveratrol was tested in models of a variety of pathologies, including stress, depression, ethanol-induced toxicity, chronic fatigue, stroke, and neurodegenerative diseases [151]. In a cellular AD-model of hippocampal tissue treated with Aβ, Rege et al. found that resveratrol protected cells from oxidative damage, by attenuating lipid peroxidation and increasing levels of endogenous antioxidants, such as glutathione reductase, catalase, superoxide dismutase, tocopherol, and ascorbic acid. Interestingly, resveratrol also amplified the expression of memory-related synaptic proteins, attenuating this AD-symptom in vitro [152].

In an in vitro model of stroke using oxygen-glucose deprivation/reoxygenation (OGD/R), resveratrol increased NSCs proliferation and survival in a concentration-dependent manner [153]. Furthermore, in rats subjected to a focal cerebral ischemia, resveratrol was found to be a plasticity inducer through the upregulation of Nrf-2 and HO-1 [154].

Resveratrol was tested in patients with mild to moderate AD in a double-blind, placebo-controlled trial, being safe and well-tolerated. This phytochemical and its metabolites were detected in the CNS, verifying they cross the blood-brain barrier. Unexpectedly, resveratrol increased the brain volume loss, but this effect was not associated with cognitive or functional decline. To determine whether resveratrol would be beneficial in AD treatments, alone or in combination with other drugs, larger clinical studies are required [155].

## 8. Conclusions

The discovery of the existence of stem cell niches in the adult human brain has stimulated the research of the therapeutic potential of the regenerative medicine for the treatment of CNS-pathologies. Replacement of damaged neural tissues by healthy cells would be a breakthrough for the therapy of many acute, chronic, or degenerative diseases. However, adult neurogenesis is a complex process that involves a myriad of mediators, such as transcription and epigenetic factors, signalling pathways, growth factors, neurotransmitters, and hormones. Current efforts are devoted to characterize the relevance, extent and significance of neurogenesis in the adult brain, and to determine whether the pharmacological stimulation of the underlying processes can address one of the toughest challenges in therapeutics: the possibility of replacing damaged neuronal tissue by functional de novo neuronal bodies fully integrated in the CNS-circuitry. In this effort, small molecules may play a central role as potential candidates to become brain-restoring drugs and/or pharmacological tools that intervene at different levels of neurogenic pathways. However, a number of challenges derived from the scarcity of neurogenic niches in adult human brain and the complexity of the involved processes need to be addressed before neurogenic-stimulating small-molecules can be used in the common medical practice. 

The good news are related to the diversity of targets that seem to be involved in adult neurogenesis, as well as the diversity of chemical scaffolds able to intervene in these processes. Thus, there is a vast field for the design and synthesis of new neurogenic small molecules as potential drugs for neurological diseases or as pharmacological tools for unravelling molecular mechanisms of adult neurogenesis.

## Abbreviations and IUPAC Names of Cited Drugs

Aβ	beta-amyloid peptide
*N*-Acetylserotonin	*N*-(2-(5-hydroxy-1*H*-indol-3-yl)ethyl)acetamide
AChE	Acetylcholinesterase
AD	Alzheimer’s disease
Andrographolide	3-[2-(Decahydro-6-hydroxy-5-hydroxymethyl-5,8*a*-dimethyl-2-methylene-1-napthalenyl)ethylidene]dihydro-4-hydroxy-2(3*H*)-furanone
Agomelatine	*N*-[2-(7-Methoxynaphthalen-1-yl)ethyl]acetamide
ALS	Amyotrophic lateral sclerosis
AR-A014418	1-(4-Methoxybenzyl)-3-(5-nitrothiazol-2-yl)urea
AREs	Antioxidant response elements
BDNF	Brain-derived neurotrophic factor
BIO	6-Bromoindirubin-3’-oxime
BrdU	Bromodeoxyuridine
BuChE	Butylcholinesterase
Buspirone	8-[4-(4-Pyrimidin-2-ylpiperazin-1-yl)butyl]-8-azaspiro[4,5]decane-7,9-dione
CaMKIV	Calcium/calmodulin-dependent protein kinase IV
CNS	Central nervous system
Curcumin	(1*E*,6*E*)-1,7-Bis(4-hydroxy-3-methoxyphenyl)-1,6-heptadiene-3,5-dione
DG	Dentate gyrus
DHEA	Dehydroepiandrosterone
ERK	Extracellular signal-regulated kinase
Fluoxetine	*N*-Methyl-3-phenyl-3-[4-(trifluoromethyl)phenoxy]propan-1-amine
Fluvoxamine	2-{[(*E*)-{5-Methoxy-1-[4-(trifluoromethyl)phenyl]pentylidene}amino]oxy}ethanamine
GDNF	Glial cell-derived neurotrophic factor
GPCRs	G-protein-coupled receptors
GSK-3β	Glycogen synthase kinase-3β
HO-1	Heme oxygenase-1
5-HT	Serotonin
5-HTRs	Serotonergic receptors
Ketanserin	3-{2-[4-(4-Fluorobenzoyl)piperidin-1-yl]ethyl}quinazoline-2,4(1*H*,3*H*)-dione
LTP	Long-term potentiation
Luzindole	*N*-(2-(2-Benzyl-1*H*-indol-3-yl)ethyl)acetamide
MeHg	Methylmercury
Melatonin	*N*-(2-(5-Methoxy-1*H*-indol-3-yl)ethyl)acetamide
5-MeOT	5-Methoxytryptamine
MTRs	Melatonergic receptors
Myristic acid	Tetradecanoic acid
NAD	Nicotinamide adenine dinucleotide
NAMPT	Nicotinamide phosphoribosyl transferase
NE-100	4-Methoxy-3-(2-phenylethoxy)-*N*,*N*-dipropylbenzeneethanamine
NGF	Nerve-growth factor
NQO1	NAD(P)H-quinone oxidoreductase 1
Nrf2	Nuclear erythroid 2-related factor
NSCs	Neural stem-cells
OBX	Olfactory bulbectomized (mouse)
OGD/R	Oxygen-glucose deprivation/reoxygenation
8-OH-DPAT	8-Hydroxy-2-(di-*n*-propylamino)tetralin
P7C3	1-(3,6-Dibromo-9*H*-carbazol-9-yl)-3-(phenylamino)propan-2-ol
P7C3-A20	*N*-(3-(3,6-Dibromo-9*H*-carbazol-9-yl)-2-fluoropropyl)-3-methoxyaniline
Pinoline	6-Methoxy-1,2,3,4-tetrahydro-9*H*-pyrido[3,4-*b*]indole
PKB	Protein kinase B
Resveratrol	*trans*-3,5,4′-Trihydroxystilbene
Rivastigmine	(*S*)-3-[1-(Dimethylamino)ethyl]phenyl *N*-ethyl-*N*-methylcarbamate
S32006	*N*-(Pyridin-3-yl)-1*H*-benzo[*e*]indole-3(2*H*)-carboxamide
SA-4503	1-[2-(3,4-Dimethoxyphenyl)ethyl]-4-(3-phenylpropyl)piperazine
SB-415286	3-((3-Chloro-4-hydroxyphenyl)amino)-4-(2-nitrophenyl)-1*H*-pyrrole-2,5-dione
SGZ	Subgranular zone
σRs	Sigma receptors
SSRIs	Selective serotonin reuptake inhibitors
SVZ	Subventricular zone
Tideglusib	4-Benzyl-2-(naphthalen-1-yl)-1,2,4-thiadiazolidine-3,5-dione
TrkB	Tyrosine receptor kinase
Valproate	2-Propylpentanoate
WAY100635	*N*-[2-[4-(2-Methoxyphenyl)-1-piperazinyl]ethyl]-*N*-(2-pyridyl)cyclohexane-carboxamide

## References

[1] Mason C., Dunnill P. (2008). A brief definition of regenerative medicine. Regen. Med..

[2] Thiel G.T. (2007). 40 projects in stem cell research, tissue engineering, tolerance induction and more (NRP46 “Implants and Transplants” 1999–2006). Swiss Med. Wkly..

[3] Altman J. (1962). Are new neurons formed in the brains of adult mammals?. Science.

[4] Goldman S.A., Nottebohm F. (1983). Neuronal production, migration, and differentiation in a vocal control nucleus of the adult female canary brain. Proc. Natl. Acad. Sci. USA.

[5] Ponti G., Peretto P., Bonfanti L. (2008). Genesis of neuronal and glial progenitors in the cerebellar cortex of peripuberal and adult rabbits. PLoS ONE.

[6] Harman A., Meyer P., Ahmat A. (2003). Neurogenesis in the hippocampus of an adult marsupial. Brain Behav. Evol..

[7] Gould E., Reeves A.J., Graziano M.S., Gross C.G. (1999). Neurogenesis in the neocortex of adult primates. Science.

[8] Richards L.J., Kilpatrick T.J., Bartlett P.F. (1992). De novo generation of neuronal cells from the adult mouse brain. Proc. Natl. Acad. Sci. USA.

[9] Reynolds B.A., Weiss S. (1992). Generation of neurons and astrocytes from isolated cells of the adult mammalian central nervous system. Science.

[10] Gage F.H., Coates P.W., Palmer T.D., Kuhn H.G., Fisher L.J., Suhonen J.O., Peterson D.A., Suhr S.T., Ray J. (1995). Survival and differentiation of adult neuronal progenitor cells transplanted to the adult brain. Proc. Natl. Acad. Sci. USA.

[11] Palmer T.D., Takahashi J., Gage F.H. (1997). The adult rat hippocampus contains primordial neural stem cells. Mol. Cell. Neurosci..

[12] Kempermann G., Jessberger S., Steiner B., Kronenberg G. (2004). Milestones of neuronal development in the adult hippocampus. Trends Neurosci..

[13] Eriksson P.S., Perfilieva E., Bjork-Eriksson T., Alborn A.M., Nordborg C., Peterson D.A., Gage F.H. (1998). Neurogenesis in the adult human hippocampus. Nat. Med..

[14] Russo A., Gianni L., Kinsella T.J., Klecker R.W., Jenkins J., Rowland J., Glatstein E., Mitchell J.B., Collins J., Myers C. (1984). Pharmacological evaluation of intravenous delivery of 5-bromodeoxyuridine to patients with brain tumors. Cancer Res..

[15] Decimo I., Bifari F., Krampera M., Fumagalli G. (2012). Neural stem cell niches in health and diseases. Curr. Pharm. Des..

[16] Gotz M., Nakafuku M., Petrik D. (2016). Neurogenesis in the developing and adult brain—Similarities and key differences. Cold Spring Harb. Perspect. Biol..

[17] Urbán N., Guillemot F. (2014). Neurogenesis in the embryonic and adult brain: same regulators, different roles. Front. Cell. Neurosci..

[18] Kintner C. (2002). Neurogenesis in embryos and in adult neural stem cells. J. Neurosci..

[19] Ohira K., Furuta T., Hioki H., Nakamura K.C., Kuramoto E., Tanaka Y., Funatsu N., Shimizu K., Oishi T., Hayashi M. (2010). Ischemia-induced neurogenesis of neocortical layer 1 progenitor cells. Nat. Neurosci..

[20] Balu D.T., Lucki I. (2009). Adult hippocampal neurogenesis: regulation, functional implications, and contribution to disease pathology. Neurosci. Biobehav. Rev..

[21] Kempermann G., Wiskott L., Gage F.H. (2004). Functional significance of adult neurogenesis. Curr. Opin. Neurobiol..

[22] Sprick U. (1995). Functional aspects of the involvement of the hippocampus in behavior and memory functions. Behav. Brain Res..

[23] Mongiat L.A., Schinder A.F. (2014). A price to pay for adult neurogenesis. Science.

[24] Rolando C., Taylor V. (2014). Neural stem cell of the hippocampus: Development, physiology regulation, and dysfunction in disease. Curr. Top. Dev. Biol..

[25] Duman R.S., Malberg J., Nakagawa S. (2001). Regulation of adult neurogenesis by psychotropic drugs and stress. J. Pharmacol. Exp. Ther..

[26] Abdipranoto A., Wu S., Stayte S., Vissel B. (2008). The role of neurogenesis in neurodegenerative diseases and its implications for therapeutic development. CNS Neurol. Disord. Drug Targets.

[27] Liu H., Song N. (2016). Molecular mechanism of adult neurogenesis and its association with human brain diseases. J. Cent. Nerv. Syst. Dis..

[28] Winner B., Winkler J. (2015). Adult neurogenesis in neurodegenerative diseases. Cold Spring Harb. Perspect. Biol..

[29] Prenderville J.A., Kelly A.M., Downer E.J. (2015). The role of cannabinoids in adult neurogenesis. Br. J. Pharmacol..

[30] Felsenstein K.M., Candelario K.M., Steindler D.A., Borchelt D.R. (2014). Regenerative medicine in Alzheimer’s disease. Transl. Res..

[31] Huart C., Rombaux P., Hummel T. (2013). Plasticity of the human olfactory system: The olfactory bulb. Molecules.

[32] Doze V.A., Perez D.M. (2012). G-Protein-coupled receptors in adult neurogenesis. Pharmacol. Rev..

[33] Sodhi M.S., Sanders-Bush E. (2004). Serotonin and brain development. Int. Rev. Neurobiol..

[34] Oberlander T.F. (2012). Fetal serotonin signaling: Setting pathways for early childhood development and behavior. J. Adolesc. Health.

[35] Brezun J.M., Daszuta A. (1999). Depletion in serotonin decreases neurogenesis in the dentate gyrus and the subventricular zone of adult rats. Neuroscience.

[36] Mazer C., Muneyyirci J., Taheny K., Raio N., Borella A., Whitaker-Azmitia P. (1997). Serotonin depletion during synaptogenesis leads to decreased synaptic density and learning deficits in the adult rat: A possible model of neurodevelopmental disorders with cognitive deficits. Brain Res..

[37] Xing L., Son J.H., Stevenson T.J., Lillesaar C., Bally-Cuif L., Dahl T., Bonkowsky J.L. (2015). A serotonin circuit acts as an environmental sensor to mediate midline axon crossing through EphrinB2. J. Neurosci..

[38] Benninghoff J., Gritti A., Rizzi M., Lamorte G., Schloesser R.J., Schmitt A., Robel S., Genius J., Moessner R., Riederer P. (2010). Serotonin depletion hampers survival and proliferation in neurospheres derived from adult neural stem cells. Neuropsychopharmacology.

[39] Grabiec M., Turlejski K., Djavadian R. (2009). Reduction of the number of new cells reaching olfactory bulbs impairs olfactory perception in the adult opossum. Acta Neurobiol. Exp. (Wars.).

[40] Grabiec M., Turlejski K., Djavadian R.L. (2009). The partial 5-HT1A receptor agonist buspirone enhances neurogenesis in the opossum (*Monodelphis domestica*). Eur. Neuropsychopharmacol..

[41] Guidi S., Stagni F., Bianchi P., Ciani E., Giacomini A., de Franceschi M., Moldrich R., Kurniawan N., Mardon K., Giuliani A. (2014). Prenatal pharmacotherapy rescues brain development in a Down’s syndrome mouse model. Brain.

[42] Stagni F., Giacomini A., Guidi S., Ciani E., Ragazzi E., Filonzi M., de Iasio R., Rimondini R., Bartesaghi R. (2015). Long-term effects of neonatal treatment with fluoxetine on cognitive performance in Ts65Dn mice. Neurobiol. Dis..

[43] Papp M., Gruca P., Lason-Tyburkiewicz M., Willner P. (2016). Antidepressant, anxiolytic and procognitive effects of rivastigmine and donepezil in the chronic mild stress model in rats. Psychopharmacology.

[44] Islam M.R., Moriguchi S., Tagashira H., Fukunaga K. (2014). Rivastigmine improves hippocampal neurogenesis and depression-like behaviors via 5-HT1A receptor stimulation in olfactory bulbectomized mice. Neuroscience.

[45] Islam M.R., Moriguchi S., Tagashira H., Fukunaga K. (2014). Rivastigmine restores 5-HT_1A_ receptor levels in the hippocampus of olfactory bulbectomized mice. Adv. Alzheimer’s Dis..

[46] De la Fuente Revenga M., Pérez C., Morales-García J.A., Alonso-Gil S., Pérez-Castillo A., Caignard D.H., Yáñez M., Gamo A.M., Rodríguez-Franco M.I. (2015). Neurogenic potential assessment and pharmacological characterization of 6-methoxy-1,2,3,4-tetrahydro-beta-carboline (pinoline) and melatonin-pinoline hybrids. ACS Chem. Neurosci..

[47] Zlotos D.P., Jockers R., Cecon E., Rivara S., Witt-Enderby P.A. (2014). MT_1_ and MT_2_ melatonin receptors: ligands, models, oligomers, and therapeutic potential. J. Med. Chem..

[48] Pala D., Lodola A., Bedini A., Spadoni G., Rivara S. (2013). Homology models of melatonin receptors: challenges and recent advances. Int. J. Mol. Sci..

[49] Hardeland R., Cardinali D.P., Srinivasan V., Spence D.W., Brown G.M., Pandi-Perumal S.R. (2011). Melatonin—A pleiotropic, orchestrating regulator molecule. Prog. Neurobiol..

[50] Reiter R.J., Manchester L.C., Tan D.X. (2010). Neurotoxins: free radical mechanisms and melatonin protection. Curr. Neuropharmacol..

[51] Tan D.X., Manchester L.C., Esteban-Zubero E., Zhou Z., Reiter R.J. (2015). Melatonin as a potent and inducible endogenous antioxidant: synthesis and metabolism. Molecules.

[52] Pandi-Perumal S.R., BaHammam A.S., Brown G.M., Spence D.W., Bharti V.K., Kaur C., Hardeland R., Cardinali D.P. (2013). Melatonin antioxidative defense: therapeutical implications for aging and neurodegenerative processes. Neurotox. Res..

[53] Lin L., Huang Q.X., Yang S.S., Chu J., Wang J.Z., Tian Q. (2013). Melatonin in Alzheimer’s disease. Int. J. Mol. Sci..

[54] Olcese J.M., Cao C., Mori T., Mamcarz M.B., Maxwell A., Runfeldt M.J., Wang L., Zhang C., Lin X., Zhang G. (2009). Protection against cognitive deficits and markers of neurodegeneration by long-term oral administration of melatonin in a transgenic model of Alzheimer disease. J. Pineal Res..

[55] Iguichi H., Kato K.I., Ibayashi H. (1982). Age-dependent reduction in serum melatonin concentrations in healthy human subjects. J. Clin. Endocrinol. Metab..

[56] Chu J., Tu Y., Chen J., Tan D., Liu X., Pi R. (2016). Effects of melatonin and its analogues on neural stem cells. Mol. Cell. Endocrinol..

[57] Sarlak G., Jenwitheesuk A., Chetsawang B., Govitrapong P. (2013). Effects of melatonin on nervous system aging: neurogenesis and neurodegeneration. J. Pharmacol. Sci..

[58] Manda K., Reiter R.J. (2010). Melatonin maintains adult hippocampal neurogenesis and cognitive functions after irradiation. Prog. Neurobiol..

[59] López L.C., Escames G., López A., García J.A., Doerrier C., Acuña-Castroviejo D. (2010). Melatonin, neurogenesis, and aging brain. Open Neuroendocrinol. J..

[60] Ramírez-Rodríguez G., Klempin F., Babu H., Benítez-King G., Kempermann G. (2009). Melatonin modulates cell survival of new neurons in the hippocampus of adult mice. Neuropsychopharmacology.

[61] Zhang X.J., Liu L.L., Jiang S.X., Zhong Y.M., Yang X.L. (2011). Activation of the zeta receptor 1 suppresses NMDA responses in rat retinal ganglion cells. Neuroscience.

[62] Ramírez-Rodríguez G., Vega-Rivera N.M., Benítez-King G., Castro-García M., Ortíz-López L. (2012). Melatonin supplementation delays the decline of adult hippocampal neurogenesis during normal aging of mice. Neurosci. Lett..

[63] Poeggeler B. (2005). Melatonin, aging, and age-related diseases. Endocrine.

[64] Tocharus C., Puriboriboon Y., Junmanee T., Tocharus J., Ekthuwapranee K., Govitrapong P. (2014). Melatonin enhances adult rat hippocampal progenitor cell proliferation via ERK signaling pathway through melatonin receptor. Neuroscience.

[65] Liu J., Somera-Molina K.C., Hudson R.L., Dubocovich M.L. (2013). Melatonin potentiates running wheel-induced neurogenesis in the dentate gyrus of adult C3H/HeN mice hippocampus. J. Pineal Res..

[66] Ramirez-Rodriguez G., Ortiz-Lopez L., Dominguez-Alonso A., Benitez-King G.A., Kempermann G. (2011). Chronic treatment with melatonin stimulates dendrite maturation and complexity in adult hippocampal neurogenesis of mice. J. Pineal Res..

[67] Jang S.W., Liu X., Pradoldej S., Tosini G., Chang Q., Iuvone P.M., Ye K. (2010). *N*-Acetylserotonin activates TrkB receptor in a circadian rhythm. Proc. Natl. Acad. Sci. USA.

[68] Iuvone P.M., Boatright J.H., Tosini G., Ye K. (2014). *N*-Acetylserotonin: Circadian activation of the BDNF receptor and neuroprotection in the retina and brain. Adv. Exp. Med. Biol..

[69] Huang E.J., Reichardt L.F. (2003). Trk receptors: Roles in neuronal signal transduction. Annu. Rev. Biochem..

[70] Chen Z., Simmons M.S., Perry R.T., Wiener H.W., Harrell L.E., Go R.C. (2008). Genetic association of neurotrophic tyrosine kinase receptor type 2 (NTRK2) With Alzheimer’s disease. Am. J. Med. Genet. B Neuropsychiatr. Genet..

[71] Tosini G., Ye K., Iuvone P.M. (2012). *N*-Acetylserotonin: Neuroprotection, neurogenesis, and the sleepy brain. Neuroscientist.

[72] Guardiola-Lemaitre B., de Bodinat C., Delagrange P., Millan M.J., Munoz C., Mocaer E. (2014). Agomelatine: Mechanism of action and pharmacological profile in relation to antidepressant properties. Br. J. Pharmacol..

[73] Pompili M., Serafini G., Innamorati M., Venturini P., Fusar-Poli P., Sher L., Amore M., Girardi P. (2013). Agomelatine, a novel intriguing antidepressant option enhancing neuroplasticity: A critical review. World J. Biol. Psychiatry.

[74] Banasr M., Soumier A., Hery M., Mocaer E., Daszuta A. (2006). Agomelatine, a new antidepressant, induces regional changes in hippocampal neurogenesis. Biol. Psychiatry.

[75] Soumier A., Banasr M., Lortet S., Masmejean F., Bernard N., Kerkerian-Le-Goff L., Gabriel C., Millan M.J., Mocaer E., Daszuta A. (2009). Mechanisms contributing to the phase-dependent regulation of neurogenesis by the novel antidepressant, agomelatine, in the adult rat hippocampus. Neuropsychopharmacology.

[76] López-Iglesias B., Pérez C., Morales-García J.A., Alonso-Gil S., Pérez-Castillo A., Romero A., López M.G., Villarroya M., Conde S., Rodríguez-Franco M.I. (2014). New melatonin-*N*,*N*-dibenzyl(*N*-methyl)amine hybrids: Potent neurogenic agents with antioxidant, cholinergic, and neuroprotective properties as innovative drugs for Alzheimer’s disease. J. Med. Chem..

[77] De la Fuente Revenga M., Fernández-Sáez N., Herrera-Arozamena C., Morales-García J.A., Alonso-Gil S., Pérez-Castillo A., Caignard D.H., Rivara S., Rodríguez-Franco M.I. (2015). Novel *N*-acetyl bioisosteres of melatonin: melatonergic receptor pharmacology, physicochemical studies, and phenotypic assessment of their neurogenic potential. J. Med. Chem..

[78] Varela-Nallar L., Inestrosa N.C. (2013). Wnt signaling in the regulation of adult hippocampal neurogenesis. Front. Cell. Neurosci..

[79] Machon O., Backman M., Machonova O., Kozmik Z., Vacik T., Andersen L., Krauss S. (2007). A dynamic gradient of Wnt signaling controls initiation of neurogenesis in the mammalian cortex and cellular specification in the hippocampus. Dev. Biol..

[80] Inestrosa N.C., Arenas E. (2010). Emerging roles of Wnts in the adult nervous system. Nat. Rev. Neurosci..

[81] Lie D.C., Colamarino S.A., Song H.J., Desire L., Mira H., Consiglio A., Lein E.S., Jessberger S., Lansford H., Dearie A.R. (2005). Wnt signalling regulates adult hippocampal neurogenesis. Nature.

[82] Jessberger S., Clark R.E., Broadbent N.J., Clemenson G.D., Consiglio A., Lie D.C., Squire L.R., Gage F.H. (2009). Dentate gyrus-specific knockdown of adult neurogenesis impairs spatial and object recognition memory in adult rats. Learn. Mem..

[83] Eom T.Y., Jope R.S. (2009). Blocked inhibitory serine-phosphorylation of glycogen synthase kinase-3alpha/beta impairs in vivo neural precursor cell proliferation. Biol. Psychiatry.

[84] Sirerol-Piquer M., Gomez-Ramos P., Hernandez F., Perez M., Moran M.A., Fuster-Matanzo A., Lucas J.J., Avila J., Garcia-Verdugo J.M. (2011). GSK3beta overexpression induces neuronal death and a depletion of the neurogenic niches in the dentate gyrus. Hippocampus.

[85] Wexler E.M., Geschwind D.H., Palmer T.D. (2008). Lithium regulates adult hippocampal progenitor development through canonical Wnt pathway activation. Mol. Psychiatry.

[86] Dastjerdi F.V., Zeynali B., Tafreshi A.P., Shahraz A., Chavoshi M.S., Najafabadi I.K., Vardanjani M.M., Atashi A., Soleimani M. (2012). Inhibition of GSK-3beta enhances neural differentiation in unrestricted somatic stem cells. Cell. Biol. Int..

[87] Silva R., Mesquita A.R., Bessa J., Sousa J.C., Sotiropoulos I., Leao P., Almeida O.F., Sousa N. (2008). Lithium blocks stress-induced changes in depressive-like behavior and hippocampal cell fate: The role of glycogen-synthase-kinase-3beta. Neuroscience.

[88] Kim A.J., Shi Y., Austin R.C., Werstuck G.H. (2005). Valproate protects cells from ER stress-induced lipid accumulation and apoptosis by inhibiting glycogen synthase kinase-3. J. Cell. Sci..

[89] Boku S., Nakagawa S., Masuda T., Nishikawa H., Kato A., Takamura N., Omiya Y., Kitaichi Y., Inoue T., Kusumi I. (2014). Valproate recovers the inhibitory effect of dexamethasone on the proliferation of the adult dentate gyrus-derived neural precursor cells via GSK-3beta and beta-catenin pathway. Eur. J. Pharmacol..

[90] Fujimura M., Usuki F. (2015). Low concentrations of methylmercury inhibit neural progenitor cell proliferation associated with up-regulation of glycogen synthase kinase 3beta and subsequent degradation of cyclin E in rats. Toxicol. Appl. Pharmacol..

[91] Fiorentini A., Rosi M.C., Grossi C., Luccarini I., Casamenti F. (2010). Lithium improves hippocampal neurogenesis, neuropathology and cognitive functions in APP mutant mice. PLoS ONE.

[92] Wang J., Tan X.F., Nguyen V.S., Yang P., Zhou J., Gao M., Li Z., Lim T.K., He Y., Ong C.S. (2014). A quantitative chemical proteomics approach to profile the specific cellular targets of andrographolide, a promising anticancer agent that suppresses tumor metastasis. Mol. Cell. Proteom..

[93] Serrano F.G., Tapia-Rojas C., Carvajal F.J., Hancke J., Cerpa W., Inestrosa N.C. (2014). Andrographolide reduces cognitive impairment in young and mature AbetaPPswe/PS-1 mice. Mol. Neurodegener..

[94] Varela-Nallar L., Arredondo S.B., Tapia-Rojas C., Hancke J., Inestrosa N.C. (2015). Andrographolide stimulates neurogenesis in the adult hippocampus. Neural Plast..

[95] Morales-García J.A., Luna-Medina R., Alonso-Gil S., Sanz-Sancristóbal M., Palomo V., Gil C., Santos A., Martínez A., Pérez-Castillo A. (2012). Glycogen synthase kinase 3 inhibition promotes adult hippocampal neurogenesis in vitro and in vivo. ACS Chem. Neurosci..

[96] Lovestone S., Boada M., Dubois B., Hull M., Rinne J.O., Huppertz H.J., Calero M., Andres M.V., Gomez-Carrillo B., Leon T. (2015). A phase II trial of tideglusib in Alzheimer’s disease. J. Alzheimer's Dis..

[97] Prati F., de Simone A., Bisignano P., Armirotti A., Summa M., Pizzirani D., Scarpelli R., Perez D.I., Andrisano V., Perez-Castillo A. (2015). Multitarget drug discovery for Alzheimer's disease: Triazinones as BACE-1 and GSK-3beta inhibitors. Angew. Chem. Int., Ed..

[98] Prati F., de Simone A., Armirotti A., Summa M., Pizzirani D., Scarpelli R., Bertozzi S.M., Perez D.I., Andrisano V., Perez-Castillo A. (2015). 3,4-Dihydro-1,3,5-triazin-2(1*H*)-ones as the first dual BACE-1/GSK-3beta fragment hits against Alzheimer’s disease. ACS Chem. Neurosci..

[99] Weissman A.D., Su T.P., Hedreen J.C., London E.D. (1988). Sigma receptors in post-mortem human brains. J. Pharmacol. Exp. Ther..

[100] Jansen K.L.R., Faull R.L.M., Dragunow M., Leslie R.A. (1991). Autoradiographic distribution of sigma receptors in human neocortex, hippocampus, basal ganglia, cerebellum, pineal and pituitary glands. Brain Res..

[101] Sha S., Qu W.J., Li L., Lu Z.H., Chen L., Yu W.F., Chen L. (2013). Sigma-1 receptor knockout impairs neurogenesis in dentate gyrus of adult hippocampus via down-regulation of NMDA receptors. CNS Neurosci. Ther..

[102] Mishina M., Ohyama M., Ishii K., Kitamura S., Kimura Y., Oda K., Kawamura K., Sasaki T., Kobayashi S., Katayama Y. (2008). Low density of sigma1 receptors in early Alzheimer's disease. Ann. Nucl. Med..

[103] Prause J., Goswami A., Katona I., Roos A., Schnizler M., Bushuven E., Dreier A., Buchkremer S., Johann S., Beyer C. (2013). Altered localization, abnormal modification and loss of function of sigma receptor-1 in amyotrophic lateral sclerosis. Hum. Mol. Genet..

[104] Klette K.L., DeCoster M.A., Moreton J.E., Tortella F.C. (1995). Role of calcium in sigma-mediated neuroprotection in rat primary cortical neurons. Brain Res..

[105] Pal A., Fontanilla D., Gopalakrishnan A., Chae Y.K., Markley J.L., Ruoho A.E. (2012). The sigma-1 receptor protects against cellular oxidative stress and activates antioxidant response elements. Eur. J. Pharmacol..

[106] Yang Z.J., Carter E.L., Torbey M.T., Martin L.J., Koehler R.C. (2010). Sigma receptor ligand 4-phenyl-1-(4-phenylbutyl)-piperidine modulates neuronal nitric oxide synthase/postsynaptic density-95 coupling mechanisms and protects against neonatal ischemic degeneration of striatal neurons. Exp. Neurol..

[107] Klouz A., Said D.B., Ferchichi H., Kourda N., Ouanes L., Lakhal M., Tillement J.P., Morin D. (2008). Protection of cellular and mitochondrial functions against liver ischemia by *N*-benzyl-*N’*-(2-hydroxy-3,4-dimethoxybenzyl)-piperazine (BHDP), a sigma1 ligand. Eur. J. Pharmacol..

[108] Baulieu E.E., Robel P. (1998). Dehydroepiandrosterone (DHEA) and dehydroepiandrosterone sulfate (DHEAS) as neuroactive neurosteroids. Proc. Natl. Acad. Sci. USA.

[109] Vermeulen A. (1995). Dehydroepiandrosterone sulfate and aging. Ann. N. Y. Acad. Sci..

[110] Sunderland T., Merril C.R., Harrington M.G., Lawlor B.A., Molchan S.E., Martinez R., Murphy D.L. (1989). Reduced plasma dehydroepiandrosterone concentrations in Alzheimer’s disease. Lancet.

[111] Moriguchi S., Yamamoto Y., Ikuno T., Fukunaga K. (2011). Sigma-1 receptor stimulation by dehydroepiandrosterone ameliorates cognitive impairment through activation of CaM kinase II, protein kinase C and extracellular signal-regulated kinase in olfactory bulbectomized mice. J. Neurochem..

[112] Moriguchi S., Shinoda Y., Yamamoto Y., Sasaki Y., Miyajima K., Tagashira H., Fukunaga K. (2013). Stimulation of the sigma-1 receptor by DHEA enhances synaptic efficacy and neurogenesis in the hippocampal dentate gyrus of olfactory bulbectomized mice. PLoS ONE.

[113] Ishima T., Nishimura T., Iyo M., Hashimoto K. (2008). Potentiation of nerve growth factor-induced neurite outgrowth in PC12 cells by donepezil: Role of sigma-1 receptors and IP3 receptors. Prog. Neuropsychopharmacol. Biol. Psychiatry.

[114] Tsai S.Y., Hayashi T., Harvey B.K., Wang Y., Wu W.W., Shen R.F., Zhang Y., Becker K.G., Hoffer B.J., Su T.P. (2009). Sigma-1 receptors regulate hippocampal dendritic spine formation via a free radical-sensitive mechanism involving Rac1xGTP pathway. Proc. Natl. Acad. Sci. USA.

[115] Tsai S.Y., Pokrass M.J., Klauer N.R., Nohara H., Su T.P. (2015). Sigma-1 receptor regulates tau phosphorylation and axon extension by shaping p35 turnover via myristic acid. Proc. Natl. Acad. Sci. USA.

[116] Miller B.R., Hen R. (2015). The current state of the neurogenic theory of depression and anxiety. Curr. Opin. Neurobiol..

[117] Hayashi T., Su T.P. (2004). Sigma-1 receptor ligands: Potential in the treatment of neuropsychiatric disorders. CNS Drugs.

[118] Hindmarch I., Hashimoto K. (2010). Cognition and depression: the effects of fluvoxamine, a sigma-1 receptor agonist, reconsidered. Hum. Psychopharmacol..

[119] Moriguchi S., Sakagami H., Yabuki Y., Sasaki Y., Izumi H., Zhang C., Han F., Fukunaga K. (2015). Stimulation of sigma-1 receptor ameliorates depressive-like behaviors in CaMKIV null mice. Mol. Neurobiol..

[120] Estrada M., Pérez C., Soriano E., Laurini E., Romano M., Pricl S., Morales-García J.A., Pérez-Castillo A., Rodríguez-Franco M.I. (2016). New neurogenic lipoic-based hybrids as innovative Alzheimer’s drugs with sigma-1 agonism and beta-secretase inhibition. Future Med. Chem..

[121] Zhao Y., Liu X.Z., Tian W.W., Guan Y.F., Wang P., Miao C.Y. (2014). Extracellular visfatin has nicotinamide phosphoribosyltransferase enzymatic activity and is neuroprotective against ischemic injury. CNS Neurosci. Ther..

[122] Stein L.R., Imai S. (2014). Specific ablation of Nampt in adult neural stem cells recapitulates their functional defects during aging. EMBO J..

[123] Wang S.N., Xu T.Y., Li W.L., Miao C.Y. (2016). Targeting nicotinamide phosphoribosyltransferase as a potential therapeutic strategy to restore adult neurogenesis. CNS Neurosci. Ther..

[124] Pieper A.A., Xie S., Capota E., Estill S.J., Zhong J., Long J.M., Becker G.L., Huntington P., Goldman S.E., Shen C.H. (2010). Discovery of a proneurogenic, neuroprotective chemical. Cell.

[125] Yin T.C., Britt J.K., de Jesus-Cortes H., Lu Y., Genova R.M., Khan M.Z., Voorhees J.R., Shao J., Katzman A.C., Huntington P.J. (2014). P7C3 neuroprotective chemicals block axonal degeneration and preserve function after traumatic brain injury. Cell Rep..

[126] Latchney S.E., Jaramillo T.C., Rivera P.D., Eisch A.J., Powell C.M. (2015). Chronic P7C3 treatment restores hippocampal neurogenesis in the Ts65Dn mouse model of Down syndrome. Neurosci. Lett..

[127] De Jesus-Cortes H., Xu P., Drawbridge J., Estill S.J., Huntington P., Tran S., Britt J., Tesla R., Morlock L., Naidoo J. (2012). Neuroprotective efficacy of aminopropyl carbazoles in a mouse model of Parkinson disease. Proc. Natl. Acad. Sci. USA.

[128] Tesla R., Wolf H.P., Xu P., Drawbridge J., Estill S.J., Huntington P., McDaniel L., Knobbe W., Burket A., Tran S. (2012). Neuroprotective efficacy of aminopropyl carbazoles in a mouse model of amyotrophic lateral sclerosis. Proc. Natl. Acad. Sci. USA.

[129] Wang G., Han T., Nijhawan D., Theodoropoulos P., Naidoo J., Yadavalli S., Mirzaei H., Pieper A.A., Ready J.M., McKnight S.L. (2014). P7C3 neuroprotective chemicals function by activating the rate-limiting enzyme in NAD salvage. Cell.

[130] Kang M.I., Kobayashi A., Wakabayashi N., Kim S.G., Yamamoto M. (2004). Scaffolding of Keap1 to the actin cytoskeleton controls the function of Nrf2 as key regulator of cytoprotective phase 2 genes. Proc. Natl. Acad. Sci. USA.

[131] Johnson J.A., Johnson D.A., Kraft A.D., Calkins M.J., Jakel R.J., Vargas M.R., Chen P.C. (2008). The Nrf2-ARE pathway: An indicator and modulator of oxidative stress in neurodegeneration. Ann. N. Y. Acad. Sci..

[132] Chen Y., Xu Y., Zheng H., Fu J., Hou Y., Wang H., Zhang Q., Yamamoto M., Pi J. (2016). The role of nuclear factor E2-Related factor 2 and uncoupling protein 2 in glutathione metabolism: Evidence from an in vivo gene knockout study. Biochem. Biophys. Res. Commun..

[133] Cho H.Y. (2013). Genomic structure and variation of nuclear factor (erythroid-derived 2)-like 2. Oxid. Med. Cell. Longev..

[134] Zhao F., Wu T., Lau A., Jiang T., Huang Z., Wang X.J., Chen W., Wong P.K., Zhang D.D. (2009). Nrf2 promotes neuronal cell differentiation. Free Radic. Biol. Med..

[135] Karkkainen V., Pomeshchik Y., Savchenko E., Dhungana H., Kurronen A., Lehtonen S., Naumenko N., Tavi P., Levonen A.L., Yamamoto M. (2014). Nrf2 regulates neurogenesis and protects neural progenitor cells against Abeta toxicity. Stem Cells.

[136] Tsai Y.M., Chien C.F., Lin L.C., Tsai T.H. (2011). Curcumin and its nano-formulation: The kinetics of tissue distribution and blood-brain barrier penetration. Int. J. Pharm..

[137] Pulido-Moran M., Moreno-Fernandez J., Ramirez-Tortosa C., Ramirez-Tortosa M. (2016). Curcumin and health. Molecules.

[138] Chojnacki J.E., Liu K., Yan X., Toldo S., Selden T., Estrada M., Rodríguez-Franco M.I., Halquist M.S., Ye D., Zhang S. (2014). Discovery of 5-(4-hydroxyphenyl)-3-oxo-pentanoic acid [2-(5-methoxy-1*H*-indol-3-yl)-ethyl]-amide as a neuroprotectant for Alzheimer’s disease by hybridization of curcumin and melatonin. ACS Chem. Neurosci..

[139] Kang S.K., Cha S.H., Jeon H.G. (2006). Curcumin-induced histone hypoacetylation enhances caspase-3-dependent glioma cell death and neurogenesis of neural progenitor cells. Stem Cells Dev..

[140] Kim S.J., Son T.G., Park H.R., Park M., Kim M.S., Kim H.S., Chung H.Y., Mattson M.P., Lee J. (2008). Curcumin stimulates proliferation of embryonic neural progenitor cells and neurogenesis in the adult hippocampus. J. Biol. Chem..

[141] Vivar C. (2015). Adult hippocampal neurogenesis, aging and neurodegenerative diseases: Possible strategies to prevent cognitive impairment. Curr. Top. Med. Chem..

[142] Garcia-Alloza M., Borrelli L.A., Rozkalne A., Hyman B.T., Bacskai B.J. (2007). Curcumin labels amyloid pathology in vivo, disrupts existing plaques, and partially restores distorted neurites in an Alzheimer mouse model. J. Neurochem..

[143] Baum L., Lam C.W., Cheung S.K., Kwok T., Lui V., Tsoh J., Lam L., Leung V., Hui E., Ng C. (2008). Six-month randomized, placebo-controlled, double-blind, pilot clinical trial of curcumin in patients with Alzheimer disease. J. Clin. Psychopharmacol..

[144] Ferrieres J. (2004). The French paradox: Lessons for other countries. Heart.

[145] Sharan S., Nagar S. (2013). Pulmonary metabolism of resveratrol: In vitro and in vivo evidence. Drug Metab. Dispos..

[146] Jeon Y.O., Lee J.S., Lee H.G. (2016). Improving solubility, stability, and cellular uptake of resveratrol by nanoencapsulation with chitosan and gamma-poly (glutamic acid). Colloids Surf. B Biointerfaces.

[147] Greene L.M., Meegan M.J., Zisterer D.M. (2015). Combretastatins: More than just vascular targeting agents?. J. Pharmacol. Exp. Ther..

[148] Awasthi M., Singh S., Pandey V.P., Dwivedi U.N. (2016). Alzheimer’s disease: An overview of amyloid beta dependent pathogenesis and its therapeutic implications along with in silico approaches emphasizing the role of natural products. J. Neurol. Sci..

[149] Kumar V., Pandey A., Jahan S., Shukla R.K., Kumar D., Srivastava A., Singh S., Rajpurohit C.S., Yadav S., Khanna V.K. (2016). Differential responses of trans-resveratrol on proliferation of neural progenitor cells and aged rat hippocampal neurogenesis. Sci. Rep..

[150] Torres-Perez M., Tellez-Ballesteros R.I., Ortiz-Lopez L., Ichwan M., Vega-Rivera N.M., Castro-Garcia M., Gomez-Sanchez A., Kempermann G., Ramirez-Rodriguez G.B. (2015). Resveratrol enhances neuroplastic changes, including hippocampal neurogenesis, and memory in Balb/C mice at six months of age. PLoS ONE.

[151] Dias G.P., Cocks G., do Nascimento Bevilaqua M.C., Nardi A.E., Thuret S. (2016). Resveratrol: A potential hippocampal plasticity enhancer. Oxid. Med. Cell. Longev..

[152] Rege S.D., Geetha T., Broderick T.L., Babu J.R. (2015). Resveratrol protects beta amyloid-induced oxidative damage and memory associated proteins in H19–7 hippocampal neuronal cells. Curr. Alzheimer Res..

[153] Cheng W., Yu P., Wang L., Shen C., Song X., Chen J., Tang F., Yang Q. (2015). Sonic hedgehog signaling mediates resveratrol to increase proliferation of neural stem cells after oxygen-glucose deprivation/reoxygenation injury in vitro. Cell Physiol. Biochem..

[154] Ren J., Fan C., Chen N., Huang J., Yang Q. (2011). Resveratrol pretreatment attenuates cerebral ischemic injury by upregulating expression of transcription factor Nrf2 and HO-1 in rats. Neurochem. Res..

[155] Turner R.S., Thomas R.G., Craft S., van Dyck C.H., Mintzer J., Reynolds B.A., Brewer J.B., Rissman R.A., Raman R., Aisen P.S. (2015). A randomized, double-blind, placebo-controlled trial of resveratrol for Alzheimer disease. Neurology.

